# A drug delivery system constructed by a fusion peptide capturing exosomes targets to titanium implants accurately resulting the enhancement of osseointegration peri-implant

**DOI:** 10.1186/s40824-022-00331-0

**Published:** 2022-12-27

**Authors:** Xuewen Li, Zihao Liu, Shendan Xu, Xinying Ma, Zhezhe Zhao, Han Hu, Jiayin Deng, Cheng Peng, Yonglan Wang, Shiqing Ma

**Affiliations:** 1 Department of Stomatology, Tianjin Medical University Second Hospital, 23 Pingjiang Road, Tianjin, 300211 China; 2 School and Hospital of Stomotology, Tianjin Medical University, 12 Observatory Road, Heping District, Tianjin, 030070 China

**Keywords:** Titanium implants, Osseointegration, Exosomes, Drug delivery system

## Abstract

**Background:**

Exosomes derived from bone marrow mesenchymal stem cells (BMSC-exos) have been shown triggering osteogenic differentiation and mineralization of MSCs, but exosomes administered via bolus injections are rapidly sequestered and cleared. Therefore, we considered the implant as a new organ of patient’s body and expected to find a method to treat implant with BMSC-exos in vivo directly.

**Methods:**

A fusion peptide (PEP), as a drug delivery system (DDS) which contained a titanium-binding peptide (TBP) possessing the ability to selectively bind to the titanium surface and another peptide CP05 being able to capture exosomes expertly, is constructed to modify the titanium surface.

**Results:**

Both in vitro and in vivo experiments prove PEP retains the ability to bind titanium and exosome simultaneously, and the DDS gain the ability to target exosomes to titanium implants surface following enhancing osseointegration post-implantation. Moreover, the DDS constructed by exosomes of diverse origins shows the similar combination rate and efficiency of therapy.

**Conclusion:**

This drug delivery system demonstrates the concept that EXO-PEP system can offer an accurate and efficient therapy for treating implants with long-term effect.

**Supplementary Information:**

The online version contains supplementary material available at 10.1186/s40824-022-00331-0.

## Background

At present, 3D-printed titanium implants (3D-Ti implants) witness an increasing usage as surgical implants in the area of dental implantation, arthroplasty and spinal orthopedic surgery to repair various bone defects owing to their bioactive surface and biocompatibility [1–4]. After the implant inserted into the bone tissue [5], the titanium implant adsorbed proteins from blood and interstitial fluids onto its surface [6], translate the surface of titanium into a biological structure to which the mesenchymal stem cells (MSCs) respond. This process was called bio-translation [7]. The study proved that the 3D-printed titanium implants showed the quicker bio-translation compared with untreated, sandblasted and large-grit titanium implants, and are globally used in clinics for faster and stronger osseointegration [8]. However, the insufficient bio-translation will cause the aseptic loosing, especially when the patient’s own osteogenic ability is poor [9]. To ensure bio-translation, osseoinduction bioactive treatment of the implant is demanded [10, 11].

Bioactive molecules were explored for inducing titanium implant bio-translation by modifying on the titanium surface, including growth factors, protein, peptide, chitosan, exosome, etc. [12]. Comparing the small sized peptides and protein, the bioactive molecules with sacculus structure (such as chitosan, liposome and exosome) were not only more engineerable to carry different therapeutic ingredients according to clinical requirement, but also able to protect the contents to be metabolized quickly [13]. Among them, exosome (EXO) is the most concerned with minimal immunogenicity, which is proved by triggering less toxicities than synthetically designed nanoparticles [14]. Exosome is a nanoscale molecule packed bio-information including proteins, lipids and nucleic acids from original cell, the information enable to mediate cell-to-cell communication and induce target cells behivor [15]. It has been proved that osteogenic exosomes secreted by pre-differentiated stem cells for 10 and 15 days gained the strongest ability of osseoinduction through activating the PI3K/Akt [16] and MAPK [17] signaling pathways [18]. Exosome derived from bone marrow mesenchymal stem cells (BMSC-exos) have the potetial to trigger osteogenic differentiation and mineralization of BMSCs [19, 20]. Consequently, treating titanium implant with BMSC-exos will not only realize the bio-transtion but also induce the mineralization and deposition of extracellular matrix to form new bone binding on the implant surface, which was termed as osseointegration [21].

Once an implant implants into the patient’s body, it is not expected to be removed as far as possible, because the patient will suffer double pain and bear more economic losses if the implant is removed from the body [22]. But when uncontrolled complications occur, it is difficult to handle and repair the implant in vivo, with the long-term application effect of the titanium implant cannot be guaranteed [23]. For this reason, we considered the implant as a new organ of patient’s body and expected to find a method to treat implant in vivo directly.

The drug delivery system (DDS) was noteworthy in the treatmemt of organs in vivo [24, 25]. This system developed a nanoparticle originated in natural or synthetic substances, containing the therapeutic agents and specific ligands of the marker on the organ or cells to be treated [26]. Specific ligands identify the markable target and deliver the nanoparticle to the target organ or cells, and consequently achieve the precise administration [27]. Exosomes has been widely used in DDS therapy [28–30]. CP05 (CRHSQMTVTSRL), a peptide specifically identifies the maker transmembrane protein on the surface of exosomes (CD63) [31], successifully anchored exosomes to build DDS delivering therapeutic exosomes on the targeting tissues in vivo [32].

However, DDS has been barely used to inorganic materials [33]. Fortunately, titanium-binding peptide (TBP) was been discovered possessing the ability of binding to the titanium selectively based on the specific binding site on the titanium surface [34]. TBP is on track to construct a DDS and has a potential application prospect to target exosomes on titanium implants in vivo [35]. RPRENRGRERGL, one of TBPs, was proved to colonize on titanium in a great efficiency, which was selected as the titanium targeting motif in the DDS [36]. It was expected to achieve extraordinary targeting under the requirement of drug support therapy for implants, thereby ultimately enhancing the osseointegration of the implant and increaseing long-term success rate of titanium implantation [37].

We expect to construct a fusion peptide (PEP) linking TBP with CP05 through a linker, and the CP05 motif anchor the BMSC-exos to form a exosomes delivery system (EXO-PEP), which delivery the exosomes to the titanium surface via the TBP motif. In this way, BMSC-exos diractionally navigate onto titanium surfaces and enhance the degree of peri-implant osseointegration.

**Scheme 1:**
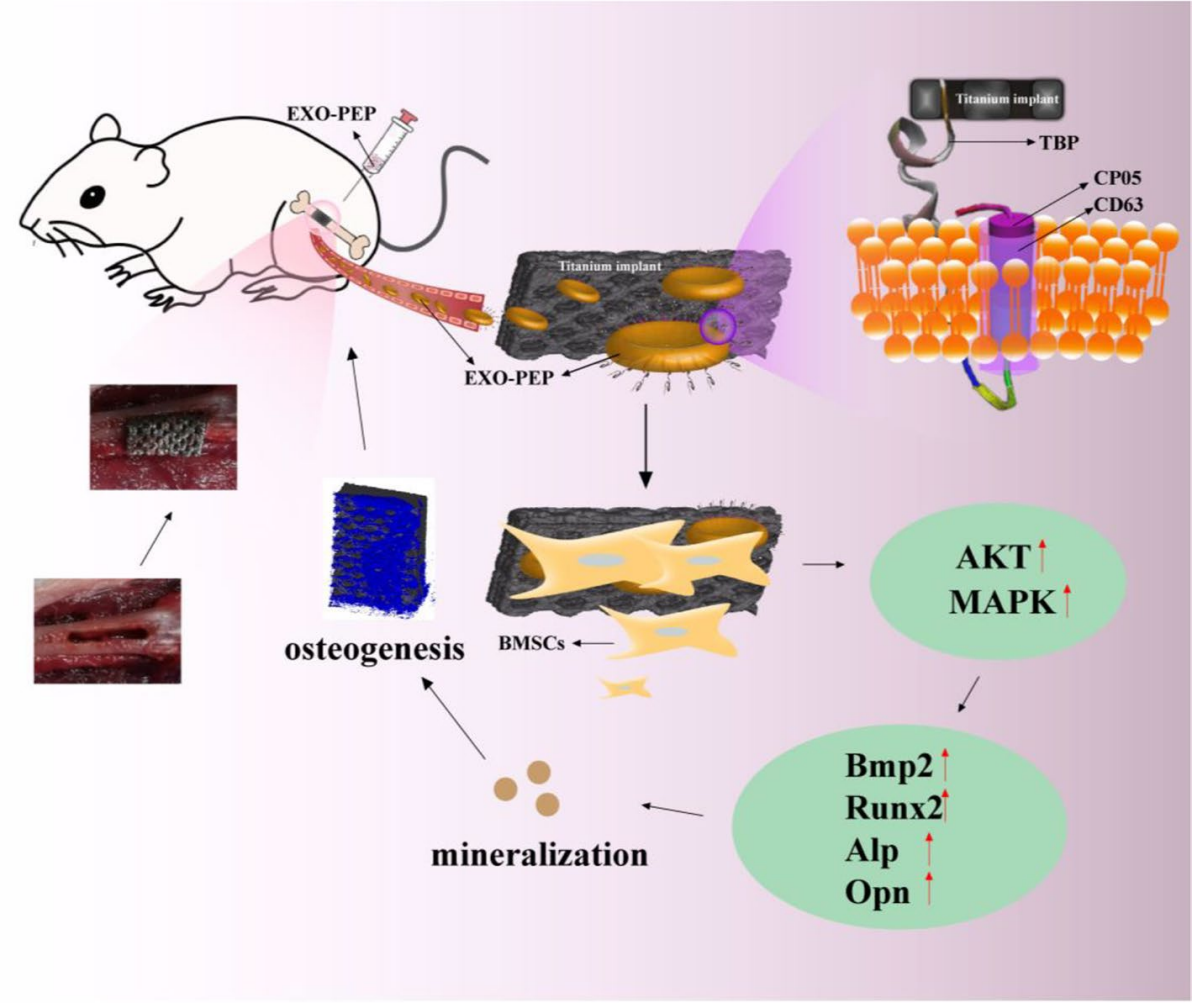
Schematic illustration of construction of EXO-PEP and 3D printing titanium plants replacement of femoral defects. EXO-PEP was oriented to the implant replaced formal bone defect in the rat model depending on the TBP motif which identified the titanium surface specifically and triggered the relative signal pathway following enhancing mineralization and osteogenesis around titanium surface

In our study, we used rats to design the femoral defects model replaced by 3D printing titanium implants (Scheme 1). We selected three TBPs with good specificity by reading the literature. The three TBPs were developed three PEPs sparely. EXO-PEP were injected into rats through caudal vein to treat implants. The orientation of the EXO-PEP was to be proved through IVIS. It was hypothesized that the EXO-PEP targeting system might achieve accurate targeting and provide peri-implant osseointegration to enhance implants stabilization.

## Methods

### Design and synthesis of fusion peptides

PEP1–3 were synthesized by the Fmoc (9-fluorenylmethyloxycarbonyl) method in Sangon Biotech Co., Ltd., Shanghai, China. Non-fluorescence peptides were provided with > 99% purity and fluorescence peptides with > 95% purity [38].

### Preparation of titanium substrates

3D titanium substrates were customizlly producted (Zhongchuhengtong Technology Development Co., Ltd., Tianjin, China). Ti discs were ultrasonically stepedlyx cleaned with acetone, ethanol (70% v/v) for 20 min, and with deionized water for 20 min.

### Cell culture

Bone mesenchyme stem cell (BMSC), umbilical cord mesenchymal stem cell (UC-MSC) and fibroblasts were all purchased from ThermoFisher scientific. All cells cultured in Dulbecco’s modified Eagle’s medium (DMEM) supplemented with 10% fetal calf serum (FBS) and 1% penicillin and streptomycin, and grown at 37 °C under a 5% CO_2_ atmosphere. For BMSC and UC-MSC, the growth medium was replaced by exosome-free DMEM continue to culture when the cells reached 70–80% confluence on the culture flask. To verify that the EXO-PEP can promote the osteogenic differentiation of BMSCs, we separately cultured BMSCs on the 3D-printed titanium discs followed by incubated with exosomes (the EXO group), EXO-PEP3 (the EXO-PEP3 group) and unfiltrated EXO-PEP3 (the EXO-PEP3 (UF) group). Un-filtered Exo-Pep (Exo-PEP (UF)) means that the unbonded peptides were not filtrated after mixing EXO and PEP during the preparation of EXO-PEP. All discs were washed by PBS for 3 times after incubation. Blank titanium discs treated with PBS (Blank) used as blank control.

### Quartz crystal microbalance (QCM)

A 9.1 MHz QCA 922A (Seiko EG&G, Japan) was charactered the binding of fusion peptides on Ti surface. The AT-cut crystal quartz sensors was coated with Ti. The peptide solution (100 μg/ml) or PBS was equilibrated in 1 mL per min at 25 ± 0.05 °C. The data of the change in resonance frequency (Δf) were recoreded. According to the Sauerbrey equation: *Δf = Δm(−n/c),* a 1 Hz decrease in frequency was equivalent to approximately 0.13 ng/cm^2^ sensor mass increase.

### X-ray photoelectron spectroscopy (XPS) and atomic force microscope (AFM)

Ti substrate analyzed for XPS was carried out under high vacuum at room temperature. A Ti substrate was incubated in 1 mg/ml peptide solution (2 mL) for 2 h at room temperature. Peptide-treated samples were rinsed extensively with PBS and ultrapure water and dried under argon gas. XPS spectra were collected using a PHI 5000 VersaProbe (ULVAC-PHI, Chigasaki, Japan) system. All binding energies were calibrated using the C 1 s (285.0 eV). The morphology of PEP recruiting of the discs were scanned at 0.5 Hz rate in air using a NanoScope VIII Multimode AFM (Bruker Corporation, USA).

### Confocal laser scanning microscope (CLSM)

For the target ability of peptides to the Ti surface, FITC labeied-PEP1–3 incubated with Ti and non-Ti specimens overnight in the 24-well plates at 4 °C sperately. For the target ability of EXO-PEP to the Ti surface, EXO-PEP1–3 was incubated with Ti specimens overnight in the 24-well cell culture plate at 4 °C. EXO was labeled by DiR and PEP was labeled by FITC. Samples washed by PBS for three times and were transferred to a glass slide, and viewed under a Laser confocal ultra-high resolution microscopy (LSM900). For exosomes uptaken, BMSCs cultured on the titanium discs. EXO or EXO-PEP treated titanium discs at 24 h after BMSCs seeded, exosomes were labeled with the DiR. Twenty-four hours later, samples were washed with PBS and fixed in 4% paraformaldehyde for 20 min at room temperature. Following PBS washing for three times the samples subsequently incubated in 6-diamidino-2-phenylindole (DAPI) solution (5 mg/ml) for 30 min at 37 °C.

### Exosome extraction

The exosome-free DMEM cultured BMSCs or UC-MSCs for 2 days was collected. The collected medium was centrifuged first at 3000 g for 20 min, second at 20,000 g for 30 min. The supernatant was collected and filtered with a 0.22-μm filter (Millex), then the supernatant was ultracentrifugated at 100,000 g for 90 min, the precipitation was resuspended by 100 μl PBS after washed with PBS. The total protein concentration of exosomes was quantified by the bicinchoninic acid (BCA) assay (Solarbio).

### Coimmunoprecipatation

Nickel Dynabeads (Solarbio) for exosomes coimmunoprecipatation analysis were preincubated with 100 μg of His-tagged PEP1–3 for 2 hours at 4 °C. Washing buffer [20 mM Na2HPO4, 500 mM NaCl, and 75 mM imidazole (pH 7.4)] were used to remove unbound peptides and eluted buffer [20 mM Na2HPO4, 500 mM NaCl, and 500 mM imidazole (pH 7.4)] were used to elute exosome lysates. by washing beads for three times. Prepared beads were incubated with 200 μg of exosome lysates for 30 min at 4 °C. After incubation, the beads were washed with washing buffer and eluted buffer. The disassociated complex (30 μl) was used for Western blot with polyclonal rabbit Alix (1:200; abcam, ab235377), CD63 (1:200; abcam, ab134045), and CD9 (1:1000; abcam, ab236630) antibodies and mouse monoclonal CD81 (1:200; abcam, ab79559).

### The distribution of PEP and EXO-PEP in vivo

AF680-labeled PEP were used for the track of peptides, the liquid of PEP (200 μl) were intravenously injected. IVIS spectrum (PerkinElmer) imaged 2, 6, 12, 24, 36, 48 h after injection separately. DiR-labeled EXO or EXO-PEP (200 μl) was used to showed the track of exosomes, the imaging was taken at 2, 6, 12, 24, 36, 48 h after injection. The organs for in vitro imaging were harvested at 12 h after injection.

### Identification of exosomes

Exosomal size distribution was measured by Nano Particle Tracking System (Malvern NS300). Morphology was visualized by a high-resolution transmission electron microscope (Hitachi HT7700). Exosome pellets for western bolt were subjected to 10% sodium dodecyl sulfate (SDS)–polyacrylamide gel electrophoresis gels and transferred to a polyvinylidene difluoride membrane. Alix, CD63, CD9 and CD81 antibodies previously metioned were used as primary antibodies to confirm the presence of exosomes. EXO-PEP system was constructed by preincubating equal exosomes and PEP overnight at 4 °C, followed by washing with PBS for five times in 2-ml ultracentrifuge tubes and filtration with 100-kDa diafiltration tube to remove unbound peptides. EXO-PEP system cultured with 4-mm aldehyde/sulfate latex beads (Invitrogen) for 15 min at room temperature, the beads were collected for CLSM and flow cytometry.

### Alizarin red staining

Cells were tested after 28 days of incubation with the EXO or EXO-PEP respectively, which first washed by PBS for 3 times and stained with 0.2% alizarin red for 30 min at 37 °C. The staining images were captured by the professional camera.

### Reverse transcription-polymerase chain reaction (RT-PCR)

RT-PCR was performed to evaluate the mRNA level of the genes related to osteogenesis. After the BMSCs were cultured for 14 days, the osteogenesis-related genes expressed by BMSCs including runt-related transcription factor 2 (Runx2), ALkaline Phosphatase (ALP), type I collagen (Col-I), osteopontin (OPN), were confirmed by RT-PCR, Glyceraldehyde-3- phosphate dehydrogenase (GAPDH) was used as a reference. Total RNA was extracted, and 200 ng of RNA template was used for a RT-PCR with a OneStep RT-PCR kit (Qiagen). The cycling conditions were 95 °C for 1 min, 57°Cfor 1 min, and 72 °C for 1 min for 25 cycles. Products were examined by electrophoresis on a 2% agarosegel.

### Immunofluorescence staining

For immunofluorescence staining, BMSCs cultured for 21 days were washed with PBS and fixed in 4% paraformaldehyde for 20 min at room temperature. Following PBS washing for three times the samples subsequently postfixed in 0.1% Triton X-100 for 5 min at room temperature. Followed by PBS washing the samples incubated with corresponding primary antibody of osteopontin (OPN) and collagen 1 (COL-1) (Abcam, Cambridge, MA, USA) overnight, following by PBS washing specimens were incubated with the secondary antibody (Abcam, Cambridge, MA, USA). The fluorescence was then imaged by the Laser confocal ultra-high resolution microscopy (LSM900).

### Western blot

For osteogenesis relative protein, antibody BMP2 (1:1000; abcam, ab284387), RUNX2 (1:1000; abcam, ab236639), ALP (1:1000; abcam, ab203106) and OPN (1:1000; abcam, ab63856) were chosen. Osteogenesis signal pathway test was used PI3K/Akt (1:1000; abcam, ab283852) and p38/MAPK14 (1:1000; abcam, ab170099) antibody. Protein extraction and Western blot were carried out as previously described. The membrane was then washed and blocked with 5% skimmed milk and probed overnight. The bound primary antibody was detected by peroxidase-conjugated goat anti-mouse IgG (Sigma-Aldrich) and the ECL Western blot analysis system (Millipore). Each experiment was performed at least three times. Statistical significance was set at 5%.

### Implant surgery

Animal experiments in this work were approved by the Animal Ethical Committee of the Academic Medical Center at the Tianjin Medical University. The Sprague-Dawley (SD) rats (250–280 g, male, 6–8 weeks) were used for osteogenesis experiments (three rat in each of the test and control groups). A driven by a handpiece was used to create a femur defect (2 mm in width，5 mm in lengthen, 4 mm in depth) on the middle of femur. For post-operation treatment, EXO/EXO-PEP (200 μl, 100 μg/ml) solution were injected through tail vein per 3 days. At 12 weeks post-operation, rats were sacrificed, and femurs were harvested and cleared of all soft tissue.

### Micro computed tomography (Micro-CT)

All femurs were kept in 75% alcohol at 4 °C prior to micro-CT scanning and histological examination. 20 circular area 50 μm-wide surrounding each implant was represented as a consistent region of interest (ROI) to quantify bone formation [39]. A high-resolution micro-CT device (Bruker Skyscan 1172, Kontich, Belgium) was used to scan the specimens at a voxel size of 13.73 μm, 100 kV, 100 μA, 360 of rotation, 0.5 mm of Al filter, a 0.7 of rotation step, 250 ms exposure. The total scanning time for the root sample was around 1 h. The tissue/implant constructs in each group were processed first into thicker sections (150 mm thick) by a microtome and then into thinner sections (150 mm thick) by grinding and polishing.

### Push-out test

A universal material testing system (Instron, High Wycombe, UK) was used to carry on the push-out test. A special holder was designed to ensure the test force was along the long axis of the implants, which terminal was fit into the cross section of the implant. All tests were processed at a loading rate of 5 mm min − 1. The load–displacement curves were recorded by computer, and the peak value of the load-displacement curve was recorded as the failure load.

### Histological analysis

For histological analysis, the femur was fixed by 10% formalin solution for 3 days and decalcified for 1 month using ethylenediaminetetraacetic acid (EDTA, 12 wt%) solution. The slices were dehydrated in gradual ethanol solution, infiltrated with xylene, and embedded in paraffin. Soft-tissue in-growth and bone formation were characterized through Van Gienson’s (VG) and Toluidine Blue (TB) staining.

### Statistics

All data were analyzed among groups using one-way ANOVA plus the least significant difference procedure (LSD) test (*p* < 0.05). The ChIP assay was analyzed by a t test (*p* < 0.05) using SPSS 19.0 software (SPSS Inc., Chicago, IL, USA).

## Result

### PEP bonded and targeted to the titanium surface specifically

#### Structure prediction of PEP

The pseudo 3D structure and amphipathic structures of PEP1–3 displayed that the clusters of secondary structure organized spatially into discrete sectors of peptides (Fig. S1A, Supporting Information). The amino acids sequence of PEP1–3 [36, 40, 41] was shown in Table 1.

**Table 1:** The amino acid sequency of each component in candidate PEPs

PEP	TBP	Linker	CP05
PEP1	ATWVSPY	PAPAP	CRHSQMTVTSRL
PEP2	RKLPDA		
PEP3	RPRENRGRERGL		

#### PEP bonded to the titanium surface successfully

**Fig. 1:**
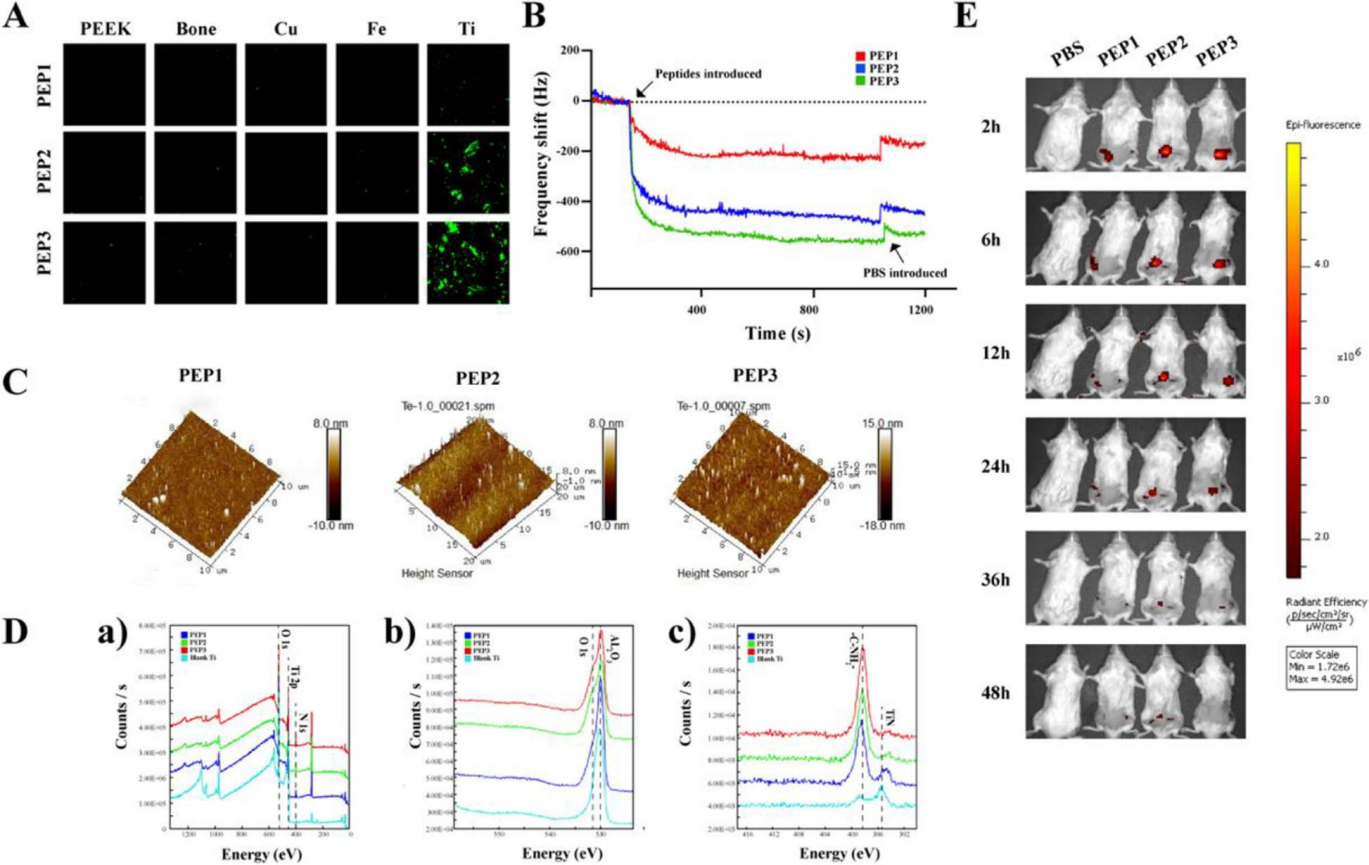
PEP bonded and targeted to the titanium surface specifically. **A** CLSM imaged PEP1–3 bonded on the Ti and non-Ti discs. **B** QCM measures the binding of PEP1–3 on Ti surfaces. Shift in frequency(∆f) versus time for exposure of the Ti sensor to PEP1–3 (100 μg/ml). At the time points indicated by the arrows, peptide solutions or phosphate buffered saline (PBS) were injected. **C** AFM images of PEP1–3 adsorbed on Ti surfaces. **D** XPS analysis of (a) total spectrum, (b) O 1 s and (c) N 1 s spectra evaluation of titanium surfaces treated with PEP1-3. **E** IVIS showed the distribution of AF680-labeled PEP1–3 in vivo

The characteristic of titanium implant surface was showed in Fig. S2. All three peptides seldom appeared on the non-Ti discs and distributed widely on the Ti discs, among that PEP1 distributed less than PEP2 and PEP3 (Fig. 1A). As shown in Fig. 1B, the frequency shift (Δf) against time was recorded when the Ti sensor was exposed to 100 μg/ml PEP solution. As the PEP introduced, all the curve of three peptides showed a significant decrease, the curve of PEP3 decreased mostly. When PBS introduced at 1000s, all of the three curves increased slightly, the least rise of which was the curve of PEP3.

Atomic force microscope (AFM) showed PEP1 was illustrated least in all groups, the density of PEP2 was as much as PEP3 (Fig. 1C). Confocal laser scanning microscope (CLSM) showed the same result (Fig. S3A, Supporting Information). The result of X-ray photoelectron spectroscopy (XPS) showed the N 1 s at 400.0 eV and O1s at 532.2 eV on the titanium discs incubated with PEP1 was lower than that with PEP2 and PEP3, and the peak in PEP2 was approximate with PEP3 (Fig. 1D).

#### PEP could target to the titanium specifically in vivo

IVIS (Fig. 1E) showed all three peptides appeared on the implant site in 0.5 hour after injection. The flurosecense density of PEP1 was less than that of PEP2 and PEP3. PEP1 almost disappeared at 12 h after injection, while PEP2 and PEP3 still existed after 36 h post-injection on the implant site.

### PEP3 adsorbed exosomes and targeted exosomes to the titanium surface

#### PEP3 bonned exosomes specifically

**Fig. 2:**
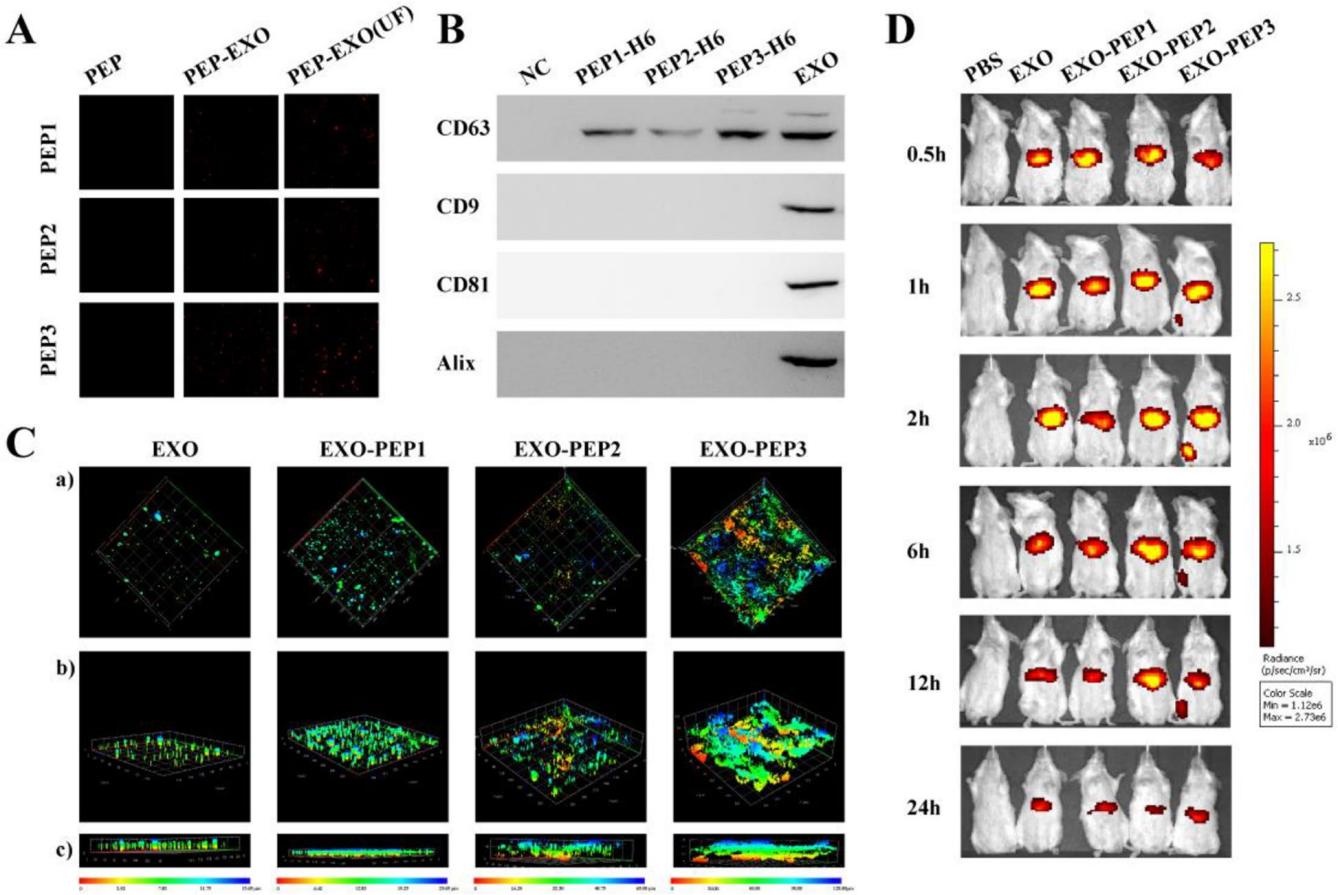
PEP bonded and targeted to the titanium surface specifically. **A** CLSM imaged EXO-PEP1–3 bonded on the titanium discs. **B** Western blot for the specific binding affinity of PEP1–3 to exosomes via CD63. **C** The depth analysis of 3D-construction DiR-labeled EXO in (a) the top view, (b) the oblique view and (c) the side view. **D** IVIS showed the distribution of DiR labeled exosomes in the EXO-PEP1–3 in vivo

BMSC-exos were extracted and incubated with polystyrene beads [42]. The beads bonding with exosomes incubated with PEP1–3 respectively and were collected for CLSM after diafiltrating unbonded peptides. The fluorescence sign of PEP3 was the highest in all three peptides and similar to that of the unfiltrated PEP3 (Fig. 2A). Western blot imaged PEP1–3 only reacted with CD63 and had no specific binds with exosomal surface markers such as CD81, CD9 and Alix, whereas a wider CD63 band was developed in the PEP3 than PEP1 and PEP2 (Fig. 2B).

#### PEP targeted exosomes to the titanium surfaces

PEP were labeled by fluorescein isothiocyanate (FITC). The fluorescence intensity of FITC in the EXO-PEP was equal to the PEP (Fig. S3B, Supporting Information), which showed the PEP fully exploited in vitro with the existence of exosomes. Figure 2C demonstrated seldom fluorescence of development inhibitor releasing coupler (DiR)-labeled exosomes existed on the titanium discs treated with exosomes only, while the fluorescence on the titanium discs treated with exosomes loaded on EXO-PEP1 was less than EXO-PEP2 and EXO-PEP3. EXO-PEP3 treated titianium discs showed the strongest fluorescence in all groups and the fluorescence uniformly appeared over the titanium surface in depth of 25 μm–100 μm according to the topography of 3D-printed titanium discs.

#### PEP captured exosomes and delivered exosomes on the titanium surface in vivo

IVIS future emphasized the important of PEP in vivo. DiR-labeled EXO-PEP were intravenously injected into rats through tail vein (Fig. 2D), tissues were imaged with IVIS (Fig. S4, Supporting Information). Without targeting peptides, exosomes were not appeared around implant. There was almost no fluorescence around femur since EXO-PEP1 and EXO-PEP2 injection, and the fluorescence concentrated on the liver, which was as same as that in the EXO group. In the situation of EXO-PEP3 injection, the fluorescence existed around implant untill 12 hour after injection and unbonded exosomes also existed in liver.

### PEP3 does not alter characteristics of exosomes

**Fig. 3:**
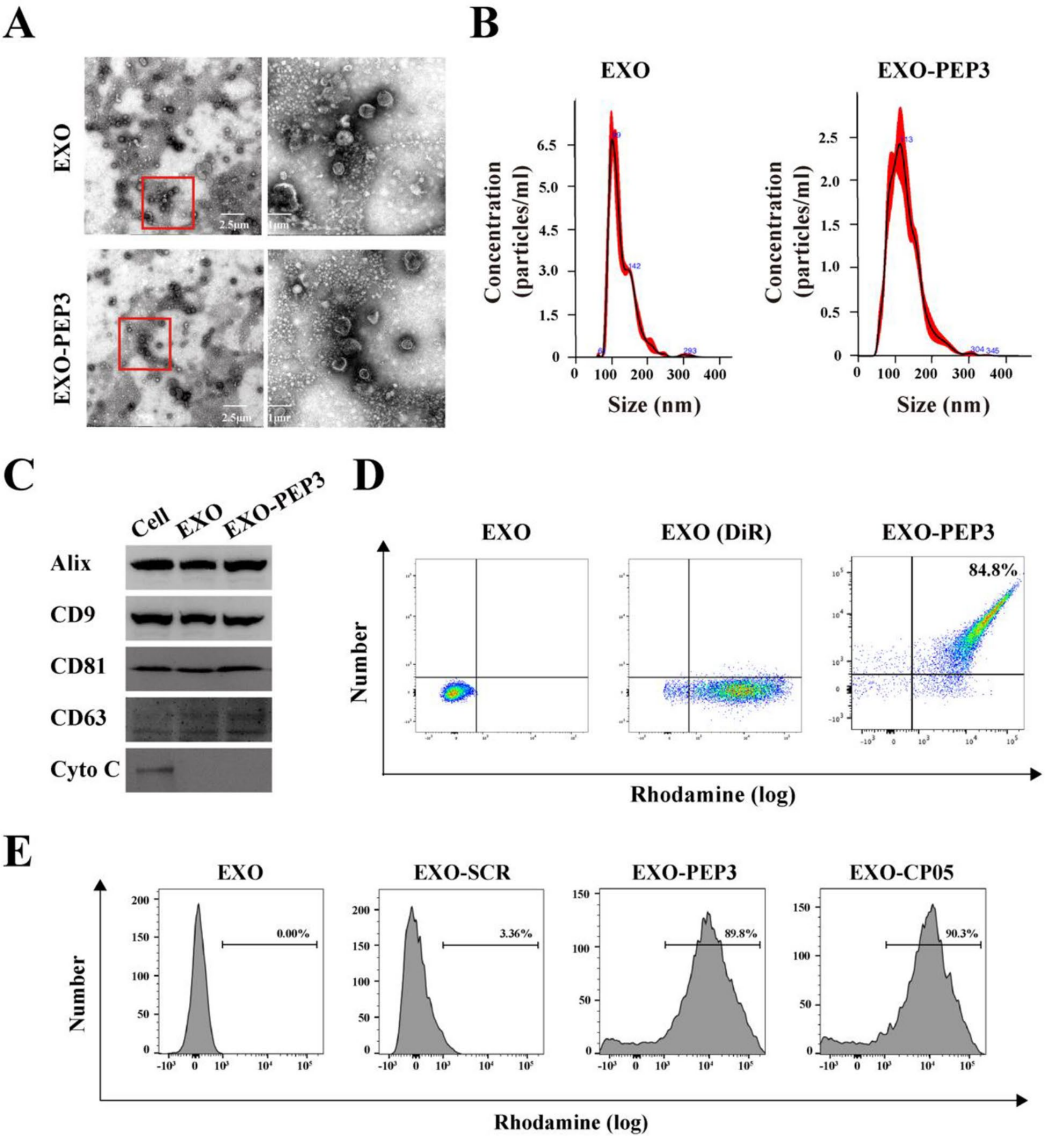
PEP3 does not alter characteristics of exosomes. **A** The shape of EXO and EXO-PEP3 was analyzed by TEM. Right panels represented the corresponding magnified boxed areas from left panels. **B** The size of EXO and EXO-PEP3 was analyzed by NTA. **C** Western blot results examined the expression of exosomal markers CD9, CD81, CD63 and Alix on the exosomes and EXO-PEP3. **D** Flow cytometry to confirm the modification efficiency of EXO with PEP3. **E** Flow cytometry to compare the modification efficiency on EXO of PEP3 and CP05. Rhodamine-labeled peptides were used

Transmission electron microscope (TEM) analysis showed that EXO exhibited specific saucer-cup shape [43], and the exosomes loaded on the EXO-PEP3 gained the same structure (Fig. 3A). Nanoparticle tracking analysis (NTA) showed the main population of sizes nanoparticles in EXO and EXO-PEP were both in a size range of 50 nm to 150 nm as the typical exosomal size [44]. The mean/mode of nanoparticles in EXO-PEP (*n* = 3) were 113 nm (Fig. 3B). Western blot (WB) analysis showed that the proteins extracted from BMSC-exos expressed the typical exosomal markers CD9, CD81, Alix and CD63, with barely detectable expression of cytosolic marker Cytochrome C (Fig. 3C) [45]. Flow cytometry demonstrated about 88.7% of DiR-labeled exosomes were modified with rhodamine-labeled PEP (Fig. 3D). The result showed no significant difference with CP05 (Fig. 3E), which resulted that the CP05 motif of PEP contained itself capability of capturing exosomes [46]. Taken together, PEP loaded on the exosomes in high proportion without affecting the structure or surface of exosomes.

### EXO-PEP enhanced osteogenesis of BMSCs thanks to targeting to the titanium surface in vitro

#### EXO-PEP for BMSCs culture was confirmed non-cytotoxic

The effect of exosome concentration on the cultured BMSCs was evaluated in CCK-8 pilot experiments. The concentration provided the highest amount of proliferation was 50 μg/ml, which used in the subsequent experiments (Fig. S6A, Supporting Information). BMSCs cultured on the titanium discs treated with exosomes grew as much as the titanium discs without special processing, which is more than that on the titanium discs treated with chitosan, magnetic nanoparticles and virus nanoparticles (Fig. S6B). It demonstrated that exosomes have non-toxicity and gain the better biocompatibility. The titnaium discs were treated with EXO-PEP3, unfiltrated EXO-PEP3 and exosomes separately, follwing washing with PBS for three times. The result of CCK-8 (Fig. S6C, Supporting Information) and AO/EB (Fig. S6D, Supporting Information) showed the BMSCs cultrualed on EXO-PEP3 (UF) group grew similiar to EXO-PEP3 group, and grew more than EXO group or blank Ti group. The BMSCs cultrualed on EXO-PEP3 and EXO-PEP3 (UF) group extended to the larger polygonal morphology, while the cells grew on the EXO group extended with short pseudopodium and the cells seeded on the blank titanium discs were still round (Fig. S6E, Supporting Information).

#### Evaluation of mineralization and osteogenic differentiation

**Fig. 4:**
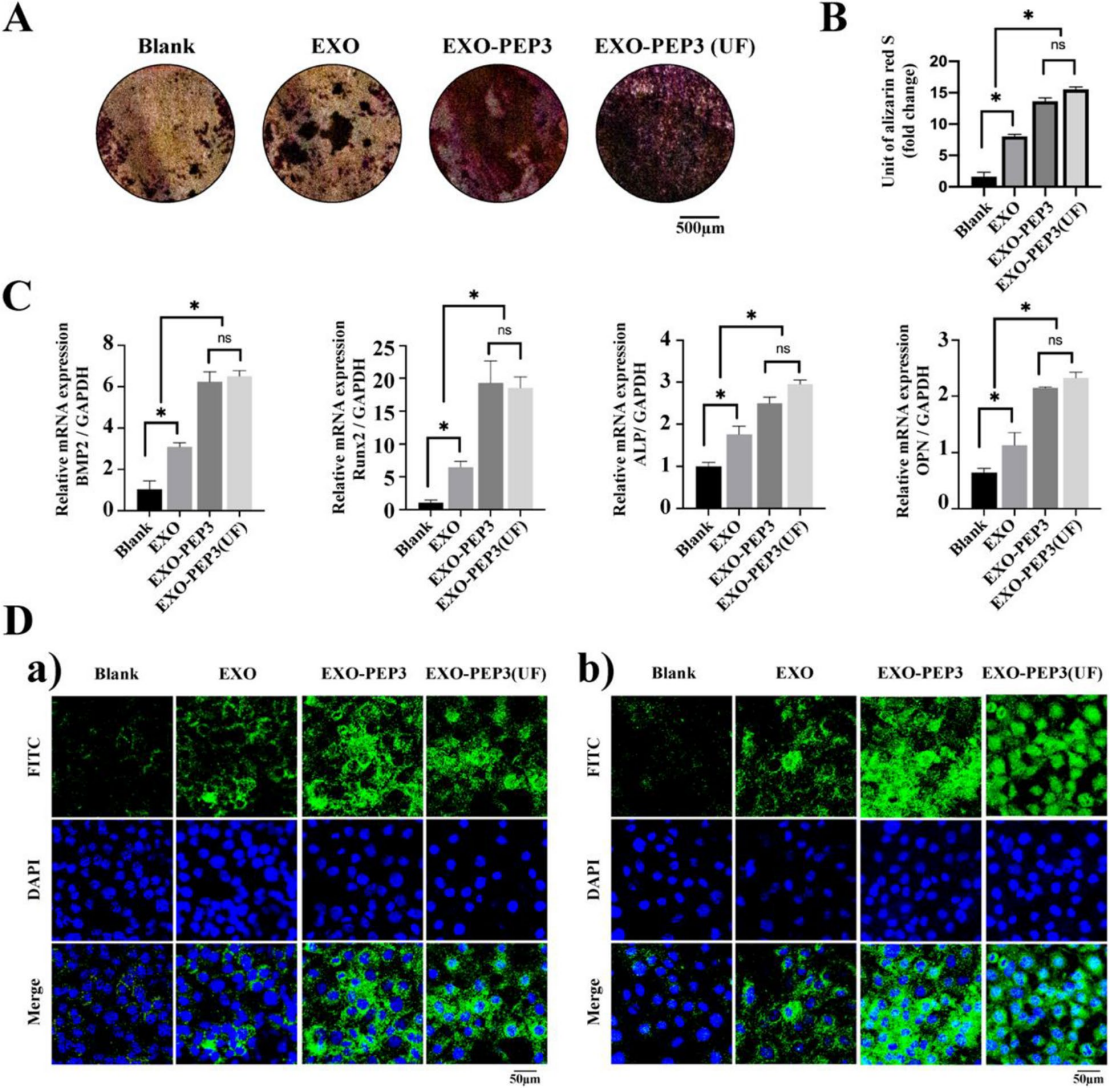
EXO-PEP3 enhanced osteogenesis of BMSCs thanks to targeting to the titanium surface in vitro. **A** The alizarin red staining determined to the mineralization of cultured BMSCs (*P* < 0.05). **B** The quantity analysis of the alizarin red staining. **C** The RNAs expression involved in the osteogenesis of the BMSCs. The significant differences are calculated by comparing each group each other. **D** Immunofluorescent staining for a) BMP2 and b) OPN in BMSCs cultured on titanium discs with different treatment. (*P* < 0.05) FITC: BMP2 or OPN; DAPI: nuclear; Merge: the merge images of FITC and DAPI

BMSCs cultured on titanium discs treated by EXO-PEP3 ingested more exosomes than EXO (*P* < 0.05) (Fig. S7, Supporting Information). At day 28, mineralized nodule formation in the EXO-PEP3 groups was substantially larger than that on the EXO and Blank groups (*p* < 0.05). The quantified amount of mineralized nodule formation on the EXO-PEP3 (UF) group was same as that on the EXO-PEP3 treated titanium (*p* < 0.05). In addition, the EXO group showed more formation of mineralized nodules as the Blank group slightly (*p* < 0.05) (Fig. 4A and B).

The gene expression of Bmp2, Runx2, Alp, and Opn was evaluated by Reverse Transcription-Polymerase Chain Reaction (RT-PCR) were shown in Fig. 4C. BMSCs cultured on the titanium treated with EXO-PEP3 presented the equal level of osteogenic gene as EXO-PEP3(UF) group, and was promoted compared to EXO and Blank groups (*p* < 0.05), while blank Ti showed the minimum level of osteogenic gene among all the groups (*p* < 0.05). The protein expression of BMP2 and OPN evaluated by immunofluorescence staining showed the same result as RT-PCR (Fig. 4D).

### EXO-PEP enhanced peri-implant osseointegration by targeting exosomes to implant

We design a femoral defects model replaced by 3D printing titanium implants with rats to conform the targeting ability of EXO-PEP3. (Fig. S8, Supporting Information). EXO and EXO-PEP were intravenously injected once every 2 days into different groups respectively. Analysis of serum biochemical showed there was no change in the concentrations of blood urea nitrogen (BUN) and creatinine (Crea) between post-injection and non-injection (Fig. S9, Supporting Information).

**Fig. 5:**
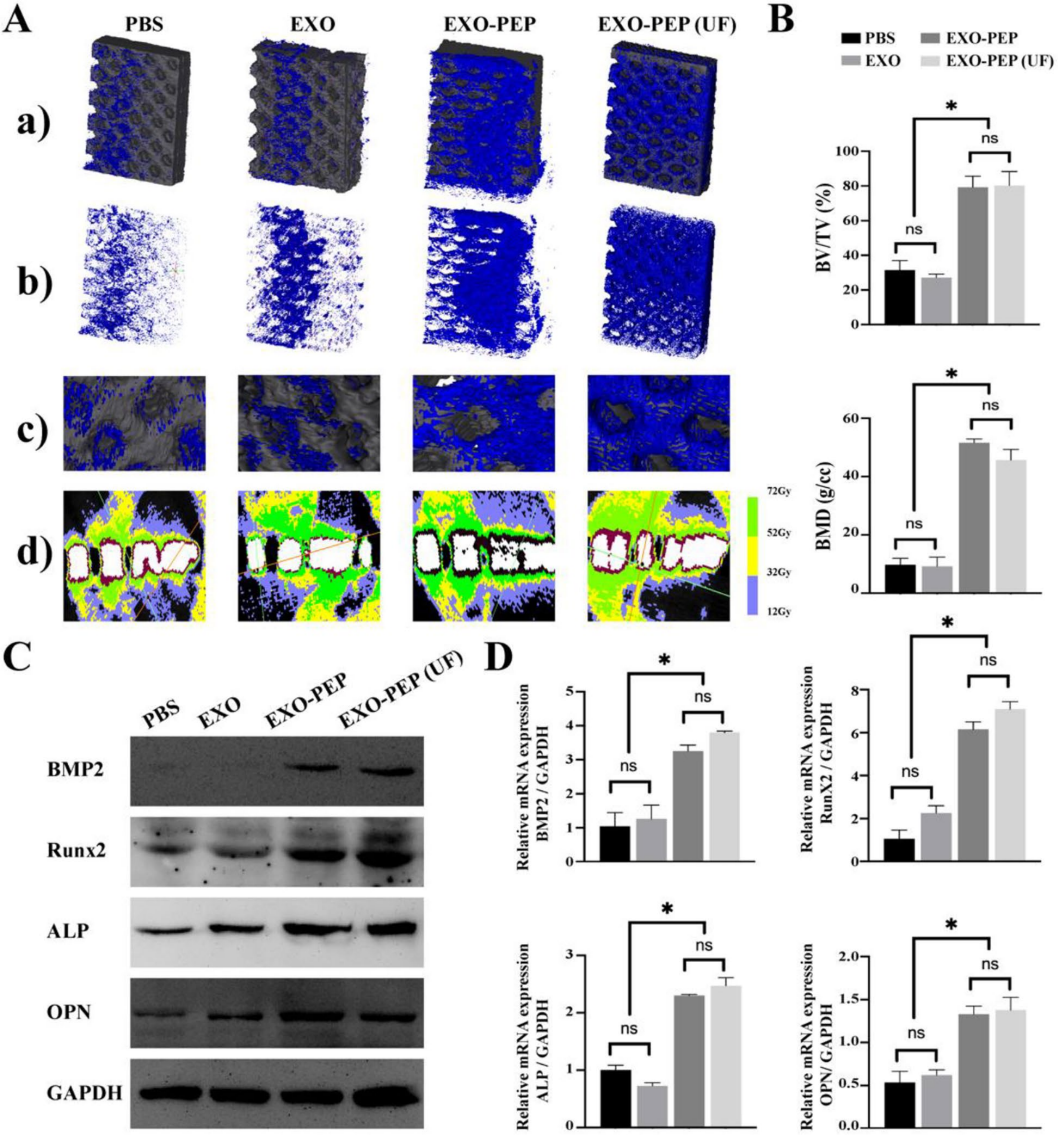
EXO-PEP3 enhanced osteogenesis peri-implants via targeting exosomes to the titanium surface in vivo. **A** Micro-CT images of reconstructed 3D models of surrounding bones (a) with or (b) without implants. (c) Local amplification of reconstructed 3D images showed the surrounding bones formation into the hole of implant. (d) The mineral density of bones surrounding the implant. **B** Quantity analysis for bone volume to total volume ratio (BV/TV) and bone mineral density (BMD). **C** Western blot analyzed the protein expression involved in the osteogenesis of the BMSCs (*p* < 0.05). **D** The RNAs expression involved in the osteogenesis of the BMSCs. The significant differences are calculated by comparing each group each other (*p* < 0.05)

**Fig. 6:**
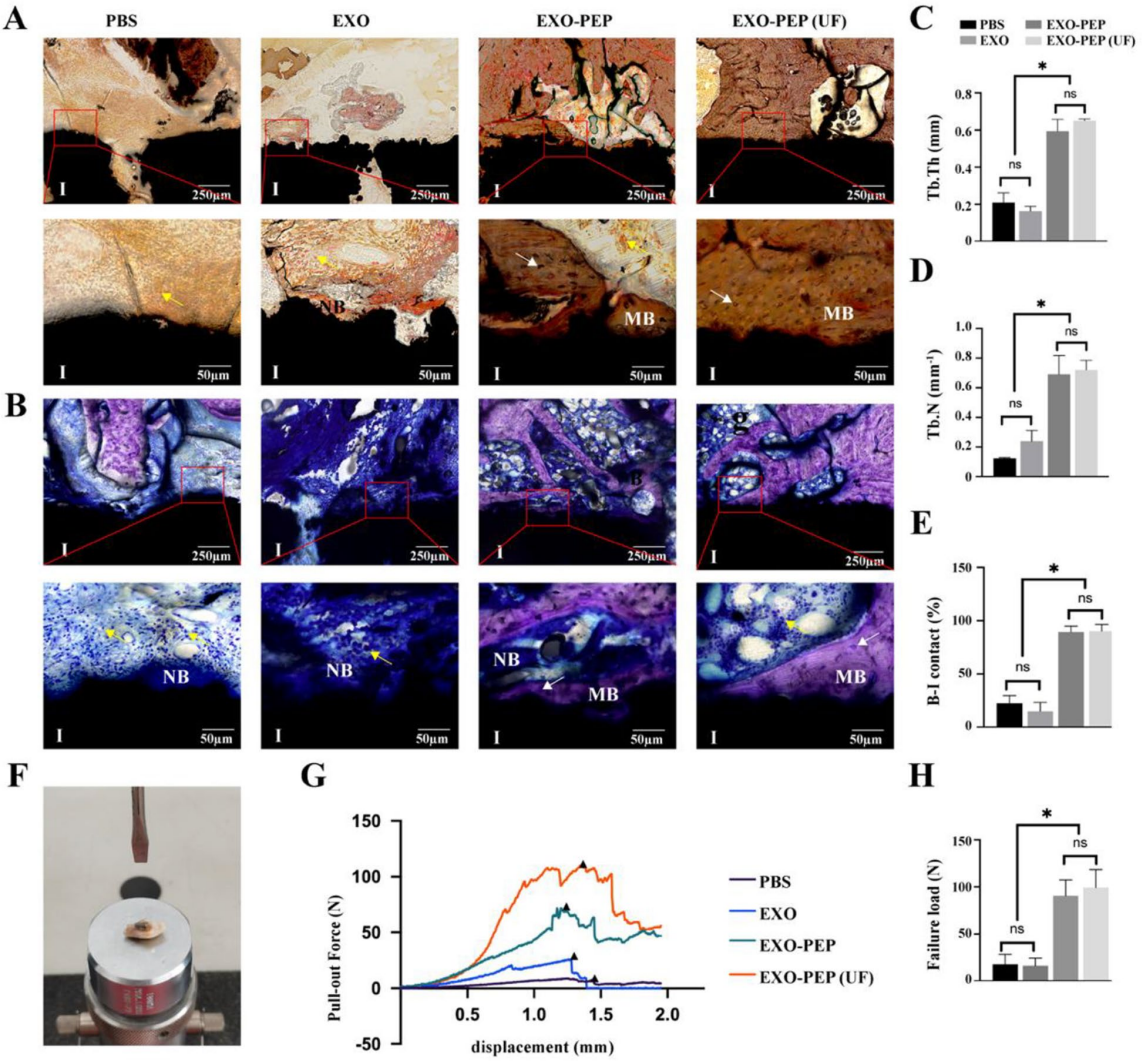
EXO-PEP3 enhanced osteointegration peri-implants through delivering exosomes to the titanium surface in vivo. **A** VG and **B** TB staining of bone defection replaced by titanium implants isolated from femur. I referred the implant, yellow arrows referred the osteoblast, white arrows referred the bone lacuna, NB referred the newly formed bone, MB referred the mature bone. Quantity analysis of histological tests for **C** trabecular bone thickness (Tb. Th.), **D** trabecular bone number (Tb. N) and **E** bone-implant contact area (B-I contact) of samples (*p* < 0.05). **F** The equipment used to test push-out test. **G** The load–displacement curves recorded by computer during test. (H) The analysis of the failure load, the peak value of the load-displacement curve (*p* < 0.05)

Micro computed tomography (Micro-CT) images of reconstructed 3D models of surrounding bones (Fig. 5A) showed that at 12 weeks after injection the bone volume surrounding implants was more in the rats treated with EXO-PEP3 than that in the rats treated with EXO. The local amplification images showed that few bone appeared on the holes of the implant in PBS and EXO groups, while bone had grown into the pores of implant in EXO-PEP3 and EXO-PEP3 (UF) group. In addition, the high mineral density bones surrounding the implant in EXO group distributed unevenly, while in the EXO-PEP3 group, high mineral density bones evenly surrounded the implant, indicating the fusion peptide could target to the titanium implant and uniformly distributed around implant carrying exosomes. The high mineral density bones surrounding the implant in EXO group was less than EXO-PEP3 group but similar to PBS group, indicating exosomes injected into rats could not arrive at the surface of titanium, while EXO-PEP3 delivered exosomes to the titanium implant successfully. The high mineral density bones surrounding the implant in the EXO-PEP3 group were approximate to the EXO-PEP3 (UF) group, suggesting the unfiltrated peptides had no influence on the PEP3 to anchor exosomes and target to the tianium in vivo. The results in bone volume to total volume ratio (BV/TV) and bone mineral density (BMD) showed the same result (Fig. 6B). The osteogenesis relative protein expressed in the EXO-PEP3 group had a twofold increase to the EXO group, which was similar to the osteogenesis gene expression, indicating exosomes oriented by PEP3 promoted peri-implant osteogenesis to a great extent.

At 12 weeks post-surgery, Toluidine Blue (TB) staining and VanGienson (VG) staining showed the new bone both formed around implants [47]. The van Gieson staining is a central histological test for establishing whether implants are in direct contact with the surrounding bones [48]. The newly formed bone contacted with titanium in a large area in EXO-PEP3 group and EXO-PEP3 (UF) (Fig. 6A). From a morphometric.

perspective, the newly formed bone on the EXO-PEP3 group multilayered surface showed a compact trabecular microstructure with higher trabecular number (Tb. N) and trabecular thickness (Tb.Th) compared to those of the microstructure on the EXO group (Fig. 6C and D). Importantly, in the EXO-PEP3 group, the implants were connected by bone almost all around with the bone-implant contact approximating to 100% (Fig. 6E). The reconstructed 3D models and section images of bone defect replaced by the implant showed the same result (Fig. S10, Supporting Information). The push-out test was utilized by a computer type tensile compression testing machine (Fig. 6F) to prove the quality of osseointegration of the implants. The result (Fig. 6G and H) showed that the implants in EXO-PEP3 (UF) groups had the failure load of 99.29 ± 19.05 N At 12 weeks after injection, which was similiar to the EXO-PEP3 groups (90.61 ± 16.85 N) (*P* > 0.05). EXO-PEP3 groups had larger failure loads than EXO groups (16.22 ± 8.27 N, *P* < 0.05). Moreover, the expression of the PI3K/Akt and p38/MAPK14 signaling pathway in the EXO-PEP3 was also higher than EXO group (*p* < 0.05) (Fig. S11, Supporting Information).

### EXO-PEP constructed with various sources of exosomes

**Fig. 7:**
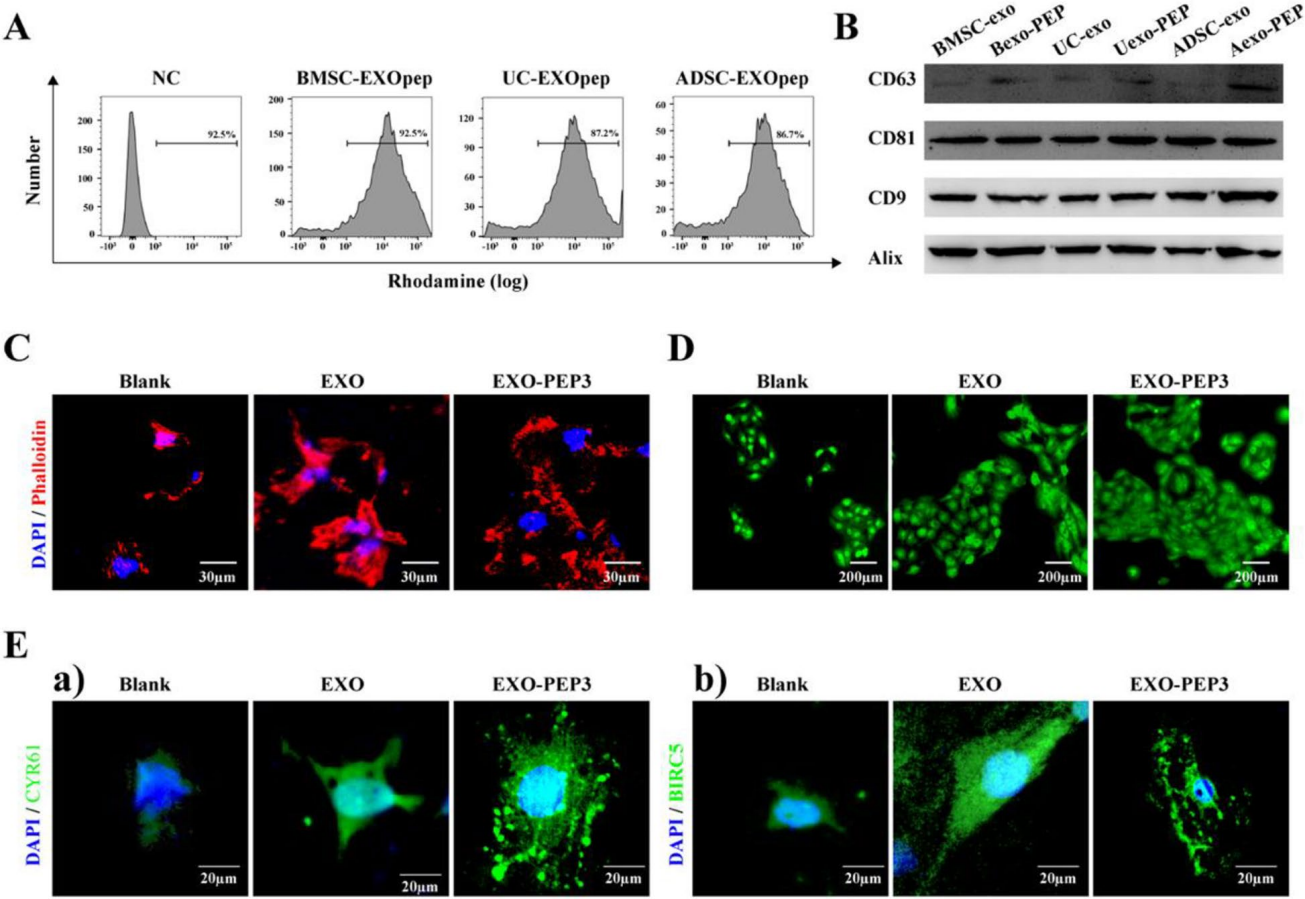
PEP3 anchored exosomes of various sources. **A** Flow cytometry to analyze the modification efficiency on PEP3 of exosomes from various derived. **B** Western blot examined the expression of exosomal markers CD9, CD81, CD63 and Alix on the various-derived exosomes with bonding by PEP3. **C** Confocal micrographs of fibroblasts on blank titanium discs and titanium diiscs treated with EXO and EXO-PEP3. Phalloidin is colored red and nuclei are colored blue. **D** AO/EB assay determined to the proliferation of fibroblasts cultured on titanium discs with different treatment. **E** Immunofluorescent staining for a) CYR61 and b) BIRC5 in fibroblasts cultured on titanium discs with different treatment. (*P* < 0.05)

Flow cytometry showed rhodamine-labeled PEP3 could bind various derived-exosomes with high binding rate (Fig. 7A). WB showed various derived exosomes still contained their typical surface signals bonded with PEP (Fig. 7B). PEP3 was incubated with UC-MSCs and constructed a UC-EXO-PEP3 system following discovering UC-EXO-PEP3 promoted the migration and proliferation of fibroblasts than that only treated with exosomes derived from UC (Fig. 7C and D). The expressed of CYR61 and BIRC5 in EXO-PEP3 was more than that in EXO (Fig. 7E).

## Discussion

The pseudo 3D structure and amphipathic structures of PEP1–3 illustrated the sections of TBP motif and CP05 motif crossed with each other in PEP1and PEP2, which may limit the both of sections. The TBP motif was independent with the CP05 motif in spatial structure in PEP3 which indicated PEP3 had the potential to gain the ability of anchoring titanium and exosomes simultaneously [49].

.It has been suggested Polyetheretherketone (PEEK) is an emerging dental implant material as widely used as titanium [50], so we choose the PEEK discs as a control groups to verify the specifical bonding of EXO-PEP with titanium [51]. The quantity of PEPs adsorbed on titanium surface suggested PEP1–3 all obtain specific bonding ability for titanium as TBP. Among all PEPs, the PEP1 showed the lightest bonding force and PEP3 realized the most peptides interacted with Ti sensor suggesting PEP3 bound with titanium in the strongest affinity [34]. A N1s speak at 400.0 eV and an O1s speak at 532.2 eV in XPS were derived from the amide groups of the peptides [52]. While a N1s speak at 395.0 eV and an O1s speak at 530.0 eV in XPS were derived from the amide groups of the Ti discs [53]. It suggested the PEP2 and PEP3 adsorbed on the Ti surface with a high density after washed by PBS, verifyied the PEP2 and PEP3 contained the functional of the motif TBP to specifically adsorb on the titanium surface. Moreover, the result of IVIS indicating PEP2 and PEP3 could orientate to titanium implant in vivo for longer.

CD63, CD81, CD9 and Alix were the typical exosomal surface markers and CP05 was a peptide identifieing the CD63 specifically, which had been successifully anchored exosomes to build DDS delivering therapeutic exosomes on the targeting tissues in vivo [54]. The result of western blot showed PEP1–3 bond with CD63 specifically via their CP05 motifs and PEP3 specifically interacted with CD63 in a stronger affinity than PEP1 and PEP2. The result of CLSM also indicated large proportion of PEP3 adsorbed exosomes and PEP1 and PEP2 adsorbed exosomes in a low proportion. It may be because the different peptides structures influenced the function of CP05 motifs that three peptides showed different binding force with CD63 [55].

When the complexes EXO-PEPs were constructed and incubated on the titanium surface, EXO-PEP1 adsorbed in the least quantity and the most connected was EXO-PEP3, suggesting PEP1 gained the low ability targeting to the titanium and PEP3 affinity as TBP3, which indicating PEP3 retained the fully titanium targeting of TBP motif and the exosomal anchoring of CP05 motif simultaneously [56]. The result of IVIS showed the similar result in vivo. The titainium targeting capacity of PEP1 in vivo was insufficient and PEP2 anchored exosomes barely. In contrary, EXO-PEP3 targets to the implant surface and the speed of exosome metabolism is retarded significantly. Therefore, It had been proved that PEP3 targeted to titanium and anchored exosomes in the best affinity, being chosen to complete the following experiment. But the area of the fluorescence was decreased apparently than that imaged in vivo, the cause of which might be that the surface area of implant was limited. Redundant EXO-PEP would remain in the vessels and be metabolized with blood circulation when there was no binding site around implant. Therefore, we chose to treat rats with multiple administration in osteogenesis experiments.

The amount of PEP in the retentates after filtration and in the unfiltrated was approximate (Fig. S5, Supporting Information). Based on this result, It is more convenient to make the PEP and exosomes into premix in order to achieve more extensive clinical application. It is only need to mix the PEP and exosome equally overnight at 4 °C before using.

EXO-PEP3 was proved to trigger the osteogenic differentiation of BMSCs and enhance the mineralization and osteogenesis on the titanium surface both in vivo and in vitro experiments. Obviously, EXO-PEP3 achieved precise administration to treat implants replaced bone defects post-surgery and enhance osteogenesis peri-implants significantly. It is worth mentioning that the osteogenesis of EXO-PEP3 group was higher than that in EXO group. Exosomes in EXO group were adsorbed on titanium surface without biological identification and washed away by PBS in a certain amount. While the exosomes in EXO-PEP3 group bonded on the titanium surface specifically so that only seldom non-bonded peptides washed away by PBS. The difference indicated that TBP retain the ability of binding titanium surface and deliver the exosomes on the titanium surface successfully.

In a previous study, it was proved that miRNAs from BMSC-exos interacted with the growth factors or receptors, like TARF6, FGF2, BMPR, BMP1, and RUNX2 to activate the PI3K/Akt and p38/MAPK14 signaling pathway for the osteogenesis of the MSCs [18]. The PI3K/Akt and MAPK signaling pathway might play a leading role in the osteogenesis of the hMSCs. We found the expression of the PI3K/Akt and p38/MAPK14 signaling pathway in the EXO-PEP3 was higher than EXO group (*p* < 0.05), which indicated that exosomes targeted to the titanium surface and triggered the osteogenic differentiation in the original way.

When the needs of patients are different, different sources of exosomes may be needed to choose for treatment. For example, it was researched that exosome derived from umbilical cord mesenchymal stem cells (UC-MSCs) facilitated wound healing and skin regeneration process via promoting the generation and migration of fibroblasts [57, 58]. We incubated PEP3 with UC-MSCs constructed a UC-EXO-PEP3 system and discovered UC-EXO-PEP3 promoted the migration and proliferation of fibroblasts than that only treated with exosomes derived from UC. CYR61 and BIRC5 are the downstream gene of TEAD critical for cell proliferation and migration [20]. The expressed of CYR61 and BIRC5 in EXO-PEP3 was more than that in EXO, which indicated EXO-PEP3 targeted UC-exos to titanium surface successfully and promote fibroblasts migration and proliferation. Therefore, PEP3 is expected to capture exosomes various derived according to different requirement and constructed a EXO-PEP system to target exosomes to the titanium implant in vivo.

Moreover, based on the background about the implant lose, the local bone microenvironment is always polluted by bacteria, virus and so on when implant lose happens [59]. It is not possible to determine whether the system can be targeted to promote bone formation after implant loosening. Therefore, we used the EXO-PEP post-operation when the implants loosing had not been occurred. It was aimed to provide early osseointegration and prevent the implanting loosing especially for patients with osteoporosis or deficient osteogenic function. So, the fusion peptide is functioned in unpolluted microenvironment and has the potential of targeted to promote bone formation before implant loosening.

Comparing the recent modifying methods of titanium implant are treated before implantation, the current application method of EXO-PEP system provides a possible method to treat implant after it inserts into patient’s body. Especially when the implant locates at the deep of the body, it is difficult to reach the implant without target system like EXO-PEP system. What’s more, there may be more than only one implant existing on the patient’s body. For example, there are 8 implants on the patient’s bone after all-on-4 implantation. It will take 8 times of injection if we treat the implants with partial treatment. However, the EXO-PEP system probably realizes to treat all implants during once administration, which will be tested in our further research. Therefore, EXO-PEP system guarantees the long-term application effect of the titanium implant, and is simple and expected to be useful clinically in the future.

## Conclusions

In conclusion, we designed a nanoparticle EXO-PEP3 to remotely target the exosomes to the 3D-printed titanium surface. PEP3 were selected from three candidate fusion peptides, which possessed the titanium targeting aility via TBP54 motif and the exosomes anchoring capability via CP05 motif. BMSC-EXO-PEP3 was successfully to anchor exosomes and target exosomes to titanium implants surface following enhancing osseointegration post-implantation. Moreover, PEP and exosomes were taken into premix, and various exosomes could be chosen according to specific clinical requirement. Therefore, the nano-exosome delivery system is expected to realize the precise targeting of therapeutic drugs to titanium implants, and used to treat implants after inserting into the bone and prolong the life-time of implants in various applications thanks to its easy synthesis and general applicability.

## Supplementary Information

**Additional file 1: Figure S1.** Pseudo-3D views and the secondary structure prediction of PEP1–3. **Figure S2.** SEM imaged the surface topography of titanium surface. **Figure S3.** A) CLSM imaged the distribution of PEP1–3. B) CLSM colocalized DiRlabeled exosomes and FITC-labeled PEP of EXO-PEP1–3. C) the fluorescence intensity was quantitated. **Figure S4.** Representative ex vivo images of A) PEPs and B) EXO-PEPs in different organs and femurs. **Figure S5.** Flow cytometry for rhodamine-labeled PEP-exosome complexes to determine the quantity of PEP diafiltrated and un-diafiltrated. **Figure S6.** A) CCK-8 assay determined most optimum concentration of EXOpep. B) CCK-8 assay determined to the cytotoxicity of different bioactive molecules. C) CCK-8 assay determined to the proliferation of BMSCs cultured on the titanium discs incubated with different treatment after 1, 2, 3, 4, 5, 6 and 7 day culture periods (*P* < 0.05). D) AO/EB assay determined to the proliferation of BMSCs. E) CLSM determined to the morphology of BMSCs. red: phalloidin; blue, DAPI. **Figure S7.** A) Characterization and B) quantity analysis of exosome uptake by hBMSCs analyzed by representative confocal microscopic images. **Figure S8.** A 3D-printed titanium implant (2 mm in thickness，5 mm in lengthen, 4 mm in width) was used to replace the femur bone defection (2 mm in width，5 mm in lengthen, 4 mm in depth) on the rat. **Figure S9.** Measurement of serum Crea and BUN in rats injected PBS, EXO, EXOPEP and EXO-PEP (UF). **Figure S10.** The 3D reconstructed models and the section images of bone defect replaced by the implant in coronal, axial and sagittal view. Purple refers bone tissue, green refers titanium implant, yellow refers the contour of bone. **Figure S11.** Western blot analysis the expression of osteogenesis signal pathway.

## Data Availability

The datasets during and/or analyzed during the current study available from the corresponding author on reasonable request.
