# Supplementary material for: A drug delivery system constructed by a fusion peptide capturing exosomes targets to titanium implants accurately resulting the enhancement of osseointegration peri-implant
Source: Biomater Res. 2022 Dec 27;26:89. doi: 10.1186/s40824-022-00331-0 (PMC9795642; doi:10.1186/s40824-022-00331-0)
Supplement: Supplementary file 1 — Additional file 1: Figure S1. Pseudo-3D views and the secondary structure prediction of PEP1–3. Figure S2. SEM imaged the surface topography of titanium surface. Figure S3. A) CLSM imaged the distribution of PEP1–3. B) CLSM colocalized DiRlabeled exosomes and FITC-labeled PEP of EXO-PEP1–3. C) the fluorescence intensity was quantitated. Figure S4. Representative ex vivo images of A) PEPs and B) EXO-PEPs in different organs and femurs. Figure S5. Flow cytometry for rhodamine-labeled PEP-exosome complexes to determine the quantity of PEP diafiltrated and un-diafiltrated. Figure S6. A) CCK-8 assay determined most optimum concentration of EXOpep. B) CCK-8 assay determined to the cytotoxicity of different bioactive molecules. C) CCK-8 assay determined to the proliferation of BMSCs cultured on the titanium discs incubated with different treatment after 1, 2, 3, 4, 5, 6 and 7 day culture periods (P < 0.05). D) AO/EB assay determined to the proliferation of BMSCs. E) CLSM determined to the morphology of BMSCs. red: phalloidin; blue, DAPI. Figure S7. A) Characterization and B) quantity analysis of exosome uptake by hBMSCs analyzed by representative confocal microscopic images. Figure S8. A 3D-printed titanium implant (2 mm in thickness，5 mm in lengthen, 4 mm in width) was used to replace the femur bone defection (2 mm in width，5 mm in lengthen, 4 mm in depth) on the rat. Figure S9. Measurement of serum Crea and BUN in rats injected PBS, EXO, EXOPEP and EXO-PEP (UF). Figure S10. The 3D reconstructed models and the section images of bone defect replaced by the implant in coronal, axial and sagittal view. Purple refers bone tissue, green refers titanium implant, yellow refers the contour of bone. Figure S11. Western blot analysis the expression of osteogenesis signal pathway. [file 40824_2022_331_MOESM1_ESM.pdf]

A Drug Delivery System Constructed by a Fusion Peptide and Captured Exosome Target to Titanium Implants Accurately Resulting the Enhancement of Osseointegration Peri-implant

Xuewen Li[1], Zihao Liu [1], Shendan Xu, Xinying Ma, Zhezhe Zhao, Han Hu, Jiayin Deng, Cheng Peng, Yonglan Wang, ***Shiqing Ma****.

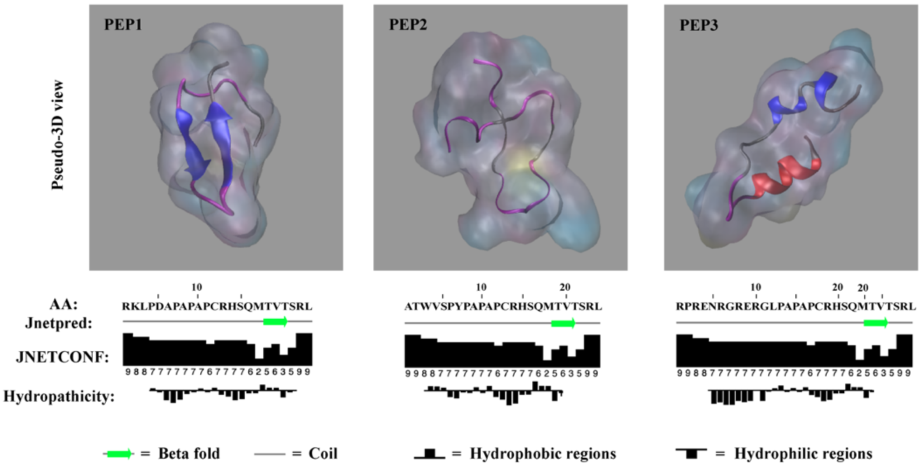

Figure S1. Pseudo-3D views and the secondary structure prediction of PEP1-3.

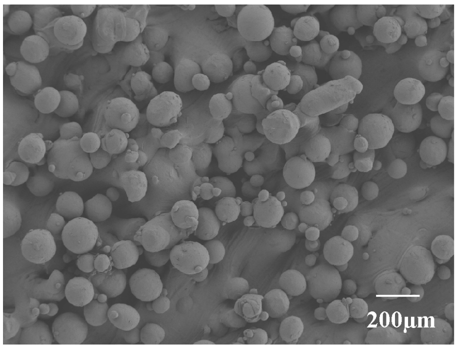

Figure S2. SEM imaged the surface topography of titanium surface.

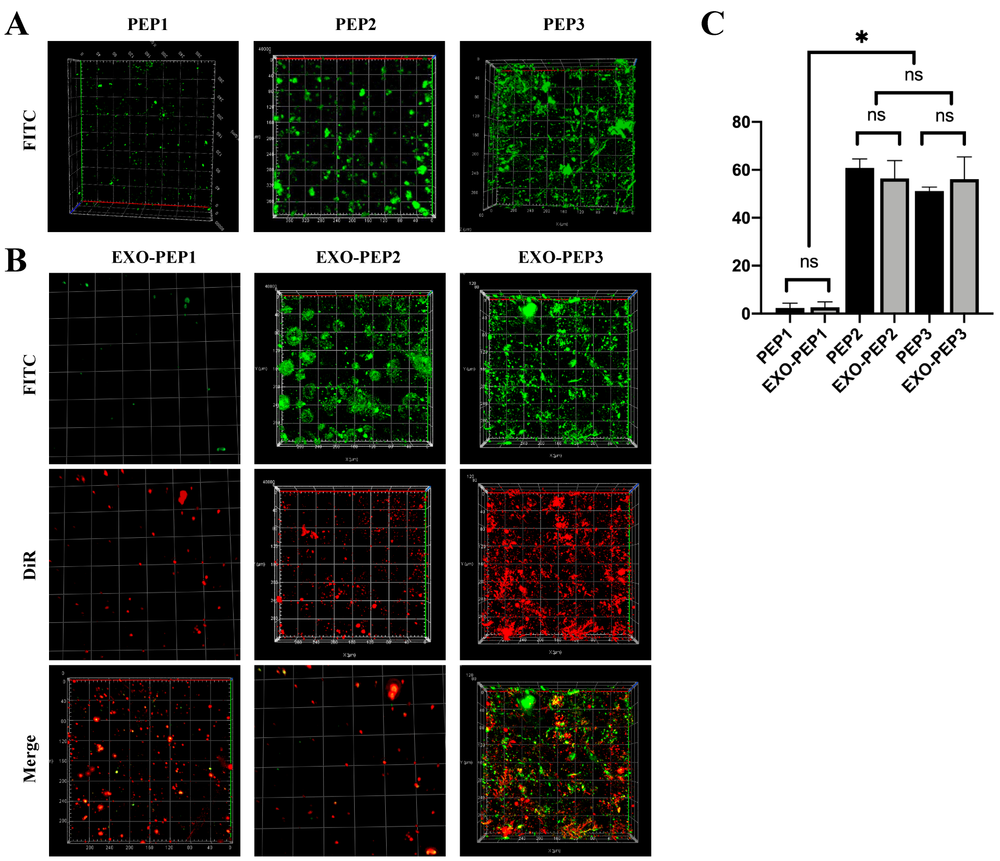

Figure S3. A) CLSM imaged the distribution of PEP1-3. B) CLSM colocalized DiR-labeled exosomes and FITC-labeled PEP of EXO-PEP1-3. C) the fluorescence intensity was quantitated.

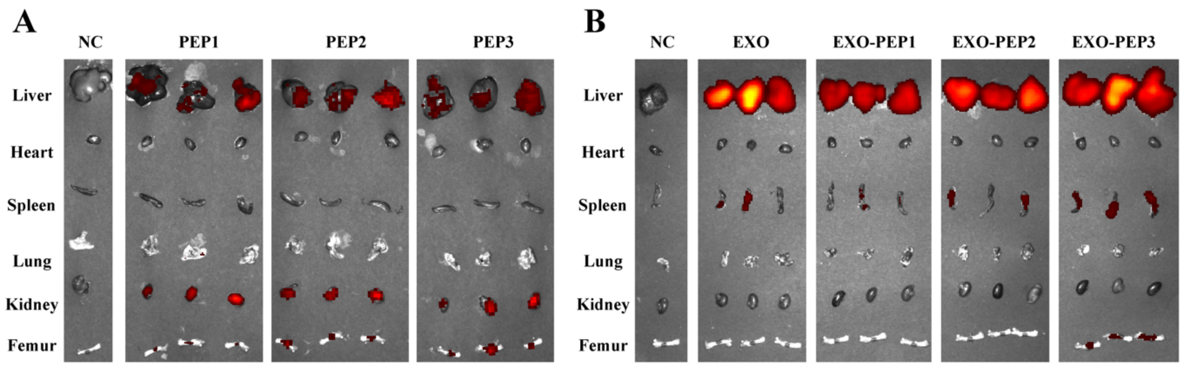

Figure S4. Representative *ex vivo* images of A) PEPs and B) EXO-PEPs in different organs and femurs.

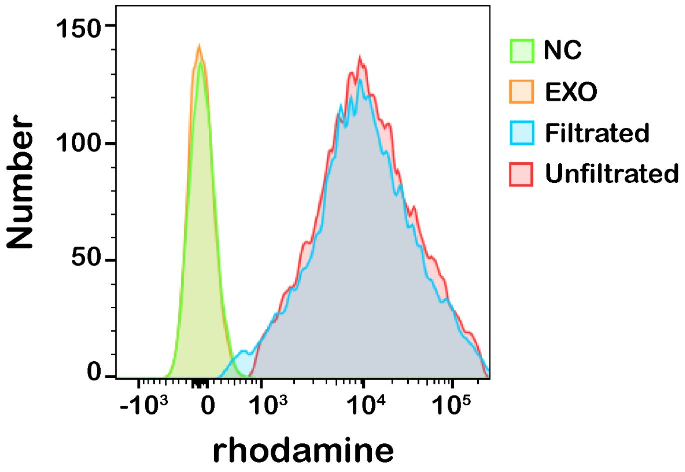

Figure S5. Flow cytometry for rhodamine-labeled PEP-exosome complexes to determine the quantity of PEP diafiltrated and un-diafiltrated.

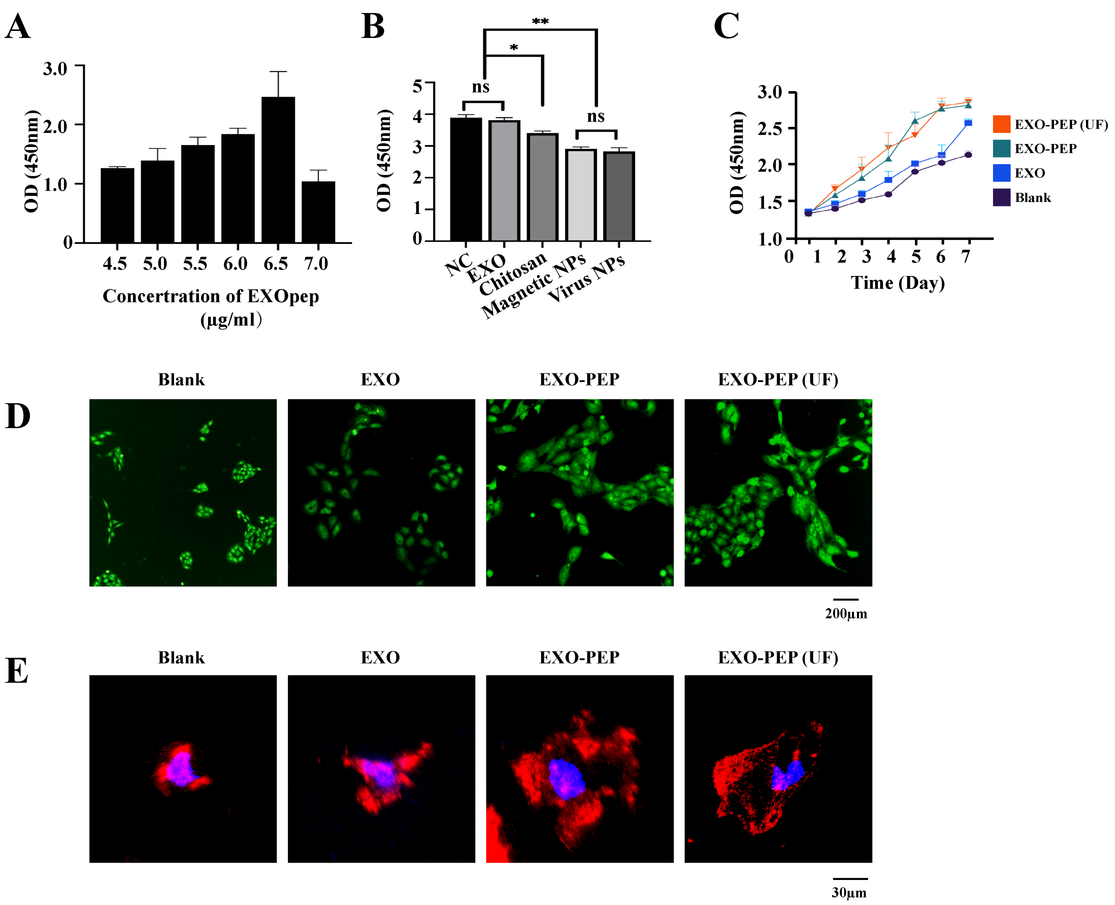

Figure S6. A) CCK-8 assay determined most optimum concentration of EXOpep. B) CCK-8 assay determined to the cytotoxicity of different bioactive molecules. C) CCK-8 assay determined to the proliferation of BMSCs cultured on the titanium discs incubated with different treatment after 1, 2, 3, 4, 5, 6 and 7 day culture periods ($P<0.05$). D) AO/EB assay determined to the proliferation of BMSCs. E) CLSM determined to the morphology of BMSCs. red: phalloidin; blue, DAPI.

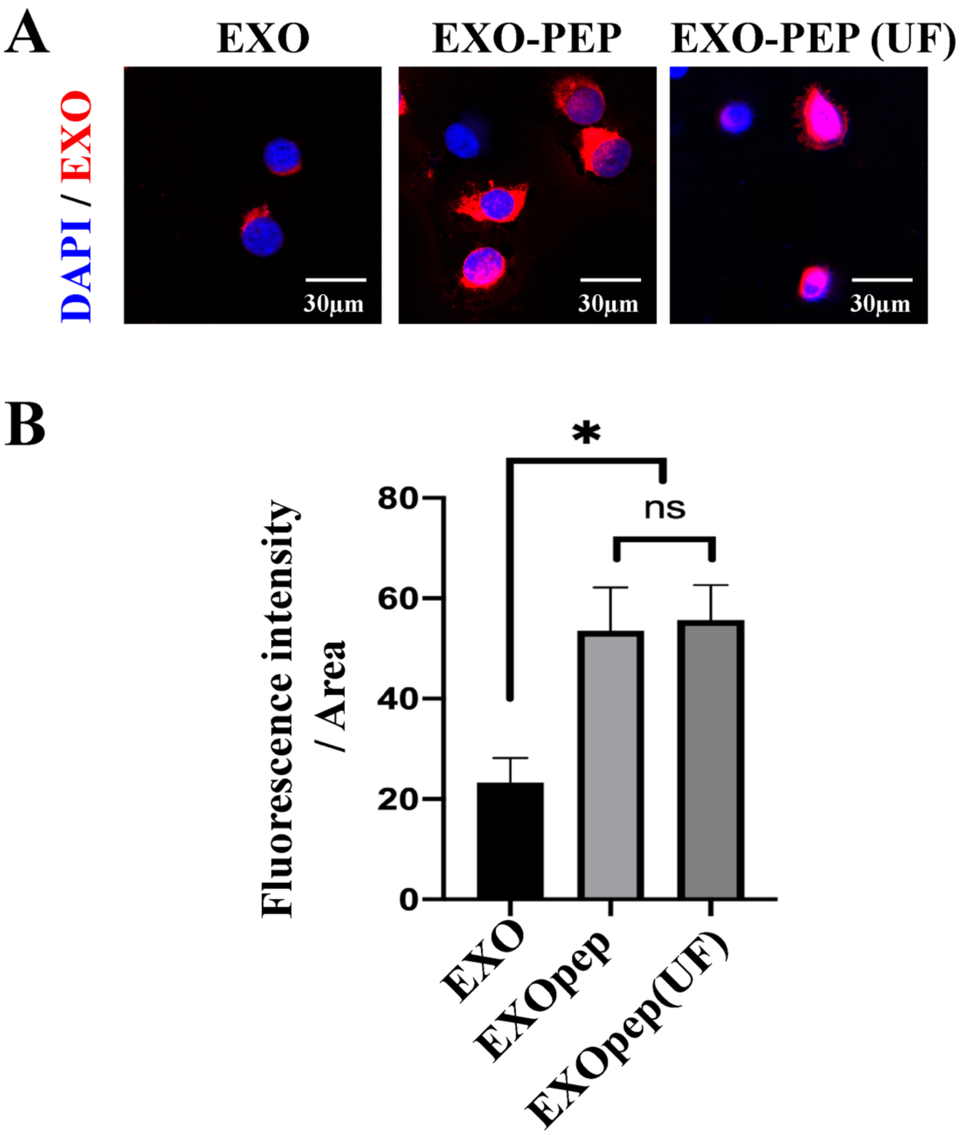

Figure S7. A) Characterization and B) quantity analysis of exosome uptake by hBMSCs analyzed by representative confocal microscopic images.

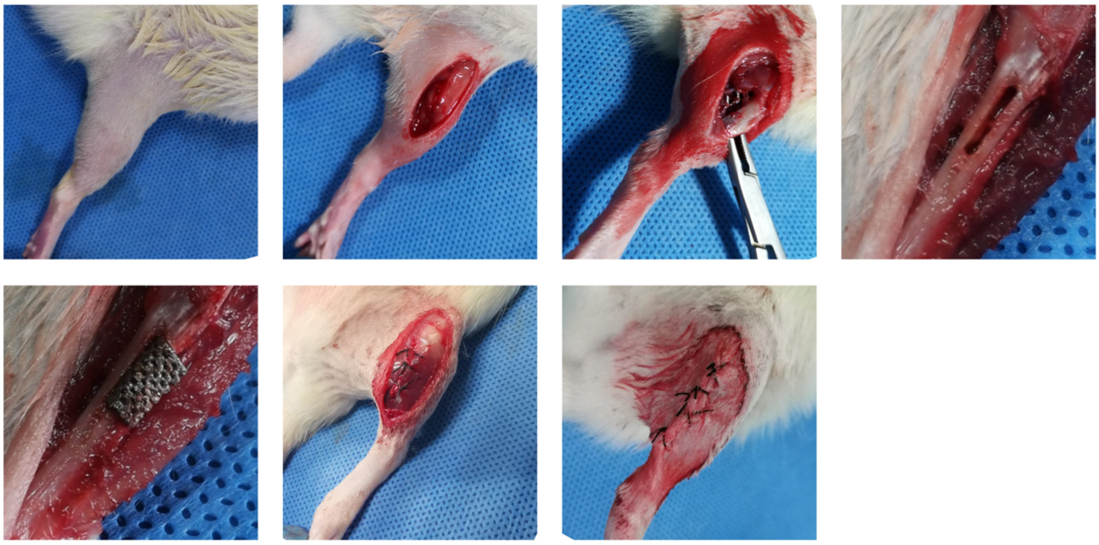

Figure S8. A 3D-printed titanium implant (2mm in thickness， 5mm in lengthen, 4mm in width) was used to replace the femur bone defection (2mm in width， 5mm in lengthen, 4mm in depth) on the rat.

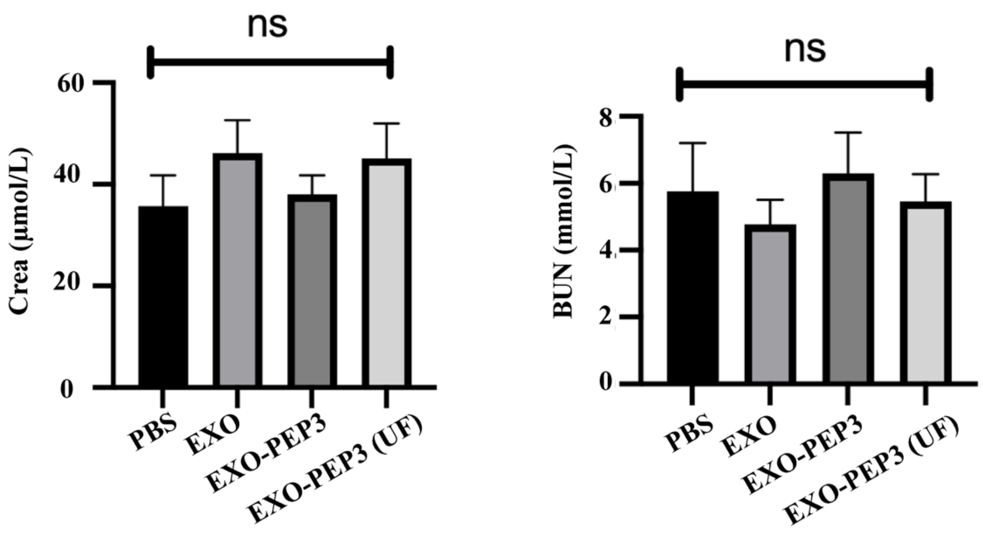

Figure S9. Measurement of serum Crea and BUN in rats injected PBS, EXO, EXO-PEP and EXO-PEP (UF).

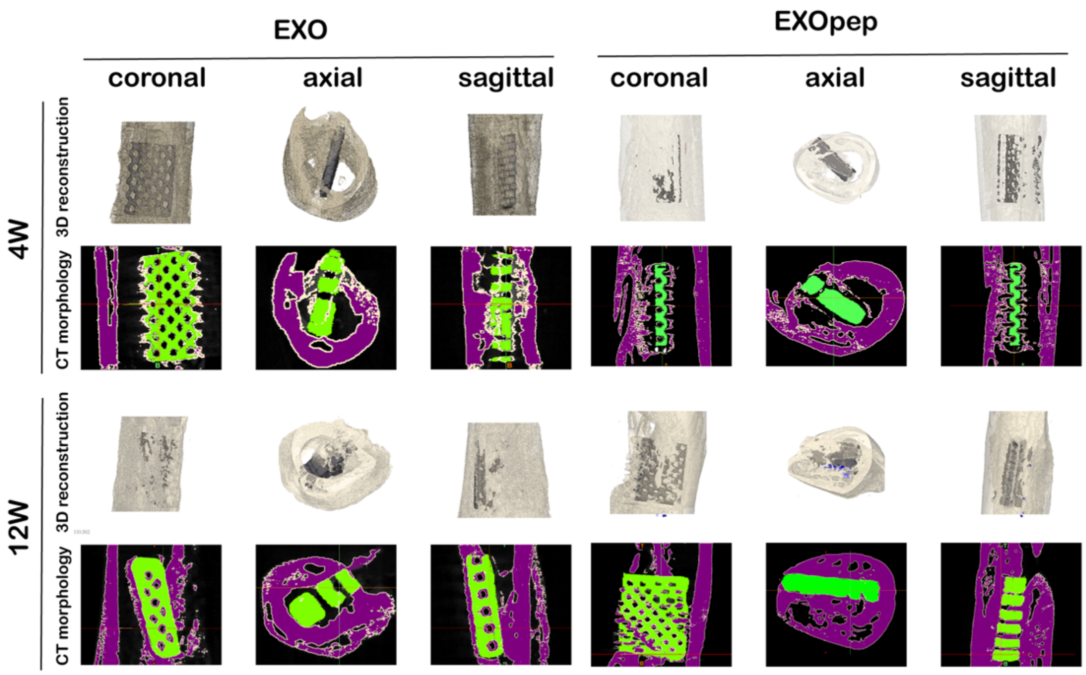

Figure S10. The 3D reconstructed models and the section images of bone defect replaced by the implant in coronal, axial and sagittal view. purple refers bone tissue, green refers titanium implant, yellow refers the contour of bone.

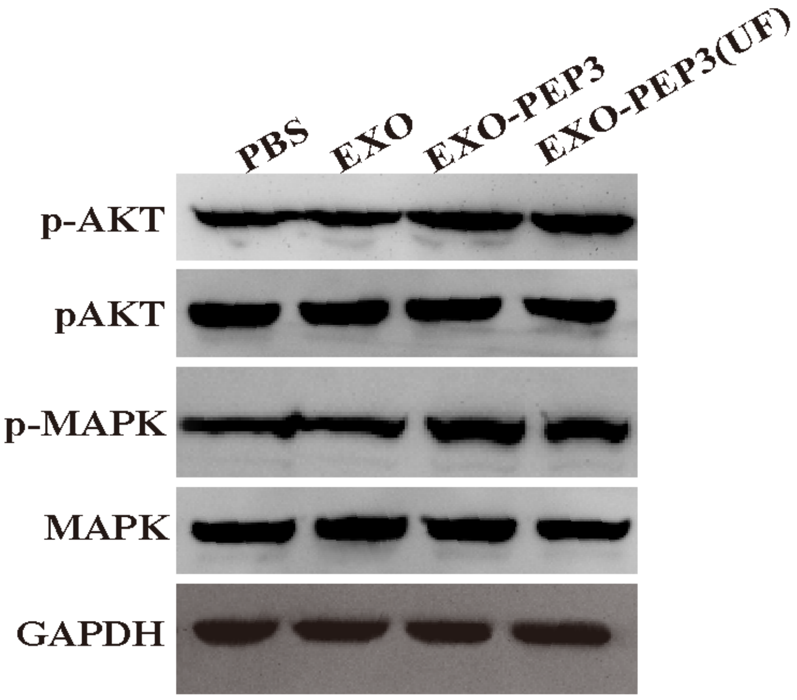

Figure S11. Western blot analysis the expression of osteogenesis signal pathway.
